# Marinacci anastomosis (reverse Martin-Gruber anastomosis): A case report

**DOI:** 10.1097/MD.0000000000025073

**Published:** 2021-04-02

**Authors:** Yu-Tai Chang, Chun-Lung Chen, Chien-Hung Lai

**Affiliations:** a Department of Physical Medicine and Rehabilitation, Taipei Medical University Hospital; b Department of Physical Medicine and Rehabilitation, Catholic Mercy Hospital, Hsinchu; c Department of Physical Medicine and Rehabilitation, School of Medicine, College of Medicine, Taipei Medical University, Taipei, Taiwan.

**Keywords:** anastomosis, Marinacci, ulnar to median

## Abstract

**Rationale:**

The incidence of Martin-Gruber anastomosis ranges from 5% to 34%, which is characterized by crossing over from the median to the ulnar nerve and innervating the first dorsal interosseous, thenar or hypothenar muscles. However, the reverse Martin-Gruber anastomosis, or Marinacci anastomosis, is far less discussed and appears in recent literature.

**Patient concerns:**

A 56-year-old man presented to the clinic of a university hospital because of left neck soreness with numbness radiating to the left lateral shoulder. The neck discomfort was aggravated while the neck rotated or tilted to the right.

**Diagnosis:**

Higher compound muscle action potential over the abductor pollicis brevis on elbow stimulation than on the wrist was found during upper limb nerve conduction velocity study. Ulnar to median anastomosis was identified.

**Intervention:**

We performed cervical spine X-ray and electrophysiological examinations and monitored the patient.

**Outcomes:**

We identified that this patient had left C5 and C6 subacute radiculopathy with active denervation and left subclinical ulnar sensory neuropathy, and verified the existence of ulnar-to-median anastomosis.

**Lessons:**

We demonstrated a pure motor ulnar-to-median anastomosis without sensory correspondence and higher CMAP over the abductor pollicis brevis on elbow stimulation of the ulnar nerve than on the wrist. The prevalence might be underestimated in a Chinese population-based published study.

## 1 Introduction

Communication between the median and ulnar nerves in the forearm has always been a challenging issue for unexperienced electromyographers who might consider them as conduction blocks. A well-known example is Martin-Gruber communication with connection crossing over from the median to the ulnar nerve and innervating the first dorsal interosseous, thenar, or hypothenar muscles,^[1,2]^ with an incidence ranging from 5% to 34%.^[3]^ The reverse Martin-Gruber communication, or Marinacci anastomosis,^[4]^ however, is far less discussed and appears in recent literature. Here, we present a case of a patient who was referred from a neurosurgeon for evaluation of right upper limb numbness, and Marinacci anastomosis was confirmed on electrophysiological study.

## 2 Ethical considerations

The case study was reviewed and approved by the Joint Institutional Review Board of Taipei Medical University (approval number: TMU-JIRB 201505042). The patient provided informed consent for the publication of the case report.

## 3 Case presentation

A 56-year-old Taiwanese man presented to the neurosurgeon clinic of a university hospital for left neck soreness with numbness radiating to the left lateral shoulder for three weeks. The neck discomfort was aggravated while the neck rotated or tilted to the right. Physical examination revealed a positive Spurling test. No muscle weakness or muscular atrophy was observed. The deep tendon reflex of the left biceps tendon was recorded as 1+. Cervical spine radiography revealed spondylosis without segmental instability. Four days later, he was referred to the Physical Medicine and Rehabilitation Department for electrophysiological examination under the impression of cervical radiculopathy. Motor conduction was studied by recording abductor pollicis brevis while stimulating median nerve at wrist and elbow, and recording abductor digiti minimi while stimulating ulnar nerve at wrist and elbow. Sensory conduction was performed by recording 2nd and 5th digital nerves when stimulating the median and ulnar nerves individually. A Medelec Synergy electromyograph (Medelec Biomedical Inc., Surrey, UK) was used for signal acquisition.

Nerve conduction showed prolonged distal latency and decreased velocity (34.6 m/s) in the ulnar sensory study. Ulnar motor study was within normal range (distal latency 2.75 m/s, velocity 61.5 m/s). On median nerve study with pickup over the abductor pollicis brevis, distal latency and velocity were both within normal range, except for the drop in amplitude between stimulation at the wrist and stimulation at the below-elbow (10.5 mv / 4.9 mV), which was compatible with the conduction block pattern. Moreover, when we left the recording electrode on the abductor pollicis brevis and stimulated the ulnar nerve at the wrist and elbow, the amplitude of the abductor pollicis brevis was higher at the elbow than at the wrist. Under the suspicion of anomalous innervations between the forearm median and ulnar nerves, we checked the sensory nerve innervation using an orthodromic study. We stimulated 3rd finger and recorded the SNAP on both the wrist and elbow of the median and ulnar nerves individually. We did not record any discrepant SNAP distribution, which suggests nerve crossing over. In the electromyography study, left C5 and C6 paraspinal muscles showed increased spontaneous activity (fibrillation and positive sharp wave). In addition, increased polyphasic waves were also found in the left biceps brachii and left extensor carpi radialis. Based on the results of the electrophysiological examination, we concluded that this patient had left C5 and C6 subacute radiculopathy with active denervation and left subclinical ulnar sensory neuropathy, and verified the existence of ulnar-to-median anastomosis.

## 4 Discussion

Marinacci^[5]^ first reported this rare anastomosis in a patient with trauma to the median nerve at the forearm. Denervation was observed in the median innervated forearm flexors, but the hand muscles were unaffected. The prevalence of Marinacci anastomosis varies according to the available literature. No case was reported in Amoiridis's work, which focused on anomalous innervation of the forearm.^[6,7]^ Studies by Rosen^[8]^ reported 5%, and Golovchinsky reported 16.7%.^[9]^ However, none of these electrophysiological studies were confirmed by anatomical data until the case report by Stančić.^[10]^ With regard to prevalence in eastern people, Kimura first reported that anastomosis from the ulnar to the median nerve was found in none of the 303 subjects.^[11]^ Later data by the same author found only two out of 150 extremities (1.3%) in 85 participants. To our knowledge, no case has been reported in the Chinese population, and our patient is the first to be identified. In our hospital electrophysiological study data bank (4116), there is no other case with a consistent pattern.

Considering the methodology for verifying Marinacci anastomosis, several points are worth mentioning. First, to avoid submaximal stimulation while stimulating the median nerve at the elbow and recording the abductor pollicis brevis, which might mimic conduction block, we rechecked the nerve conduction procedure and increased current intensity gradually to ensure that supramaximal stimulation was achieved. This procedure is similar to that of Meenakshi's work.^[12]^ Second, recording the CMAP of the ulnar-innervated thenar muscle (for example, the deep head of the flexor pollicis brevis muscle) might be erroneously regarded as ulnar-to-median anastomosis. Therefore, we checked three other university students with normal nerve conduction studies on routine median and ulnar nerves. We recorded the abductor pollicis brevis and stimulated the ulnar nerves at the elbow. Without exception, all three students’ thenar CMAPs showed an initial positive waveform. All patients showed an initial negative waveform if we stimulated the median nerve at the below-elbow. These findings are completely different from those of our patient.

Not as frequently identified and reported as Martin-Gruber communication, Marinacci anastomosis showed a reverse pattern. Knowing this normal variance has contributed to the avoidance of iatrogenic injury when performing surgical release of the carpal ligament.^[10]^ The “pseudo conduction block” pattern might erroneously be taken as a neurapraxia lesion within the forearm and would therefore spend more time searching for a non-existing lesion.

## 5 Conclusion

We demonstrated a pure motor ulnar-to-median anastomosis without sensory correspondence, which showed higher CMAP over the abductor pollicis brevis on elbow stimulation of the ulnar nerve than on the wrist. The prevalence might be underestimated in a Chinese population-based published study.^[2,8,9,11]^

## Author contributions

**Data curation:** Yu-Tai Chang, Chen MD Chun-Lung.

**Formal analysis:** Yu-Tai Chang.

**Investigation:** Yu-Tai Chang, Chen MD Chun-Lung.

**Methodology:** Yu-Tai Chang, Chen MD Chun-Lung, Chien-Hung Lai.

**Supervision:** Chien-Hung Lai.

**Visualization:** Chien-Hung Lai.

**Writing – original draft:** Yu-Tai Chang, Chen MD Chun-Lung.

**Writing – review & editing:** Yu-Tai Chang, Chien-Hung Lai.
